# The Role of Interleukin-22 and Its Receptor in the Development and Pathogenesis of Experimental Autoimmune Uveitis

**DOI:** 10.1371/journal.pone.0154904

**Published:** 2016-05-11

**Authors:** Yejin Kim, Tae Wan Kim, Yun Seong Park, Eui Man Jeong, Dong-Sup Lee, In-Gyu Kim, Hum Chung, Young-il Hwang, Wang Jae Lee, Hyeong Gon Yu, Jae Seung Kang

**Affiliations:** 1 Department of Anatomy, Seoul National University College of Medicine, Seoul, Republic of Korea; 2 Department of Ophthalmology, Seoul National University College of Medicine, Seoul, Republic of Korea; 3 Rheumatology Institute and Research for Sensory Organs Institute, Medical Research Center, Seoul National University, Seoul, Republic of Korea; 4 Department of Ophthalmology, Seoul Metropolitan Government Seoul National University, Boramae Medical Center, Seoul, Republic of Korea; 5 Department of Biochemistry and Molecular Biology, Seoul National University College of Medicine, Seoul, Republic of Korea; Boston University School of Medicine, UNITED STATES

## Abstract

IL-22 is a pro- and anti-inflammatory cytokine that is mainly produced by T cells and NK cells. Recent studies have reported the increased number of IL-22 producing T cells in patients with autoimmune noninfectious uveitis; however, the correlation between IL-22 and uveitis remains unclear. In this study, we aimed to determine the specific role of IL-22 and its receptor in the pathogenesis of uveitis. Serum concentration of IL-22 was significantly increased in uveitis patients. IL-22Rα was expressed in the retinal pigment epithelial cell line, ARPE-19. To examine the effect of IL-22, ARPE-19 was treated with recombinant IL-22. The proliferation of ARPE-19 and the production of monocyte chemoattractant protein (MCP)-1 from ARPE-19 were clearly elevated. IL-22 induced MCP-1 which facilitated the migration of inflammatory cells. Moreover, IL-22 increased the IL-22Rα expression in ARPE-19 through the activation of PI3K/Akt. Experimental animal models of uveitis induced by interphotoreceptor retinoid binding protein 1–20 (IRBP_1-20_) exhibited elevation of hyperplasia RPE and IL-22 production. When CD4^+^ T cells from the uveitis patients were stimulated with IRBP_1-20_, the production of IL-22 definitely increased. In addition, we examine the regulatory role of cysteamine, which has an anti-inflammatory role in the cornea, in uveitis through the down-regulation of IL-22Rα expression. Cysteamine effectively suppressed the IRBP_1-20_-induced IL-22Rα expression and prevented the development of IRBP_1-20_-induced uveitis in the experimental animal model. These finding suggest that IL-22 and its receptor have a crucial role in the development and pathogenesis of uveitis by facilitating inflammatory cell infiltration, and that cysteamine may be a useful therapeutic drug in treating uveitis by down-regulating IL-22Rα expression in RPE.

## Introduction

Uveitis is an inflammatory disease that develops in uvea which is the middle layer of tissue in the eye wall. It is a very serious disease because of its sudden development and quick progression. Even though it is known that bacterial infection and autoimmune responses are the major cause of disease development, early diagnosis and treatment are the most important in preventing disease progression. Experimental autoimmune uveitis (EAU) is used as an animal model to examine the pathogenesis of uveitis because it represents posterior segment intraocular inflammation in humans. EAU is organ-specific, and it is a T cell-mediated autoimmunity that is induced by immunization with retinal antigens, for example, interphotoreceptor retinoid-binding protein (IRBP) and S-Ag. Additionally, it also can be induced by adoptive transfer of retinal Ag-specific T cells [1–4]. In IRBP exposure, inflammatory factors such as Th1/Th17 cytokines increase in the affected tissues, which in turn activate cellular inflammatory responses characterized by infiltration of large numbers of inflammatory cells composed primarily of mononuclear cells [5]. Thus, EAU may be a consequence of a Th1/Th17 dominant immune response. Supporting this idea, the numbers of Th17 cells along with elevated levels of particular subsets of IL-17 have been reported to be increased in EAU, suggesting a mechanism by which Th17 cells may contribute to uveitis [6].

Th17 cells have been identified as a subset of T helper lymphocytes characterized by the production of the IL-17 cytokine family, IL-17A, IL-17B, IL-17C, IL-17D, IL-17E and IL-17F [7]. It has also been reported that Th17 cells also produce IL-22 in both mice and human [8]. Additionally, it has been newly designated as Th22 [9–11]. It is known that epithelial cells are the major target for IL-22. Moreover, it has a crucial role in the wound healing process as well as in controlling bacterial infection [12–15]. The IL-22 receptor consists of IL-22Rα and IL-10Rβ. IL-22 exerts its biological effects through binding to the heterodimer IL-22Rα/IL-10Rβ complex followed by activation of signal transducer and activator of transcription 3 (STAT3) [12, 16, 17]. It is known that IL-10Rβ is ubiquitously expressed in a variety of cell types. Moreover, the expression of IL-22Rα is restricted to epithelial cells, especially to keratinocytes in the skin and hepatocytes in the liver [12, 16–19]. Even though it is not expressed on immune cells, it has recently been reported that it is expressed on CD11b+ APC in mice through stimulation with IRBP [20].

It seems that IL-22 is closely related with autoimmune diseases such as rheumatoid arthritis (RA), Crohn’s disease, and skin inflammatory diseases by promoting inflammatory responses [21–23]. In contrast, IL-22 is also described as an anti-inflammatory cytokine family because its protective role has been reported in inflammatory bowel disease, experimental hepatitis, and experimental autoimmune myocarditis [24–27]. Recent studies have shown that IL-22 gene expression was increased in patients with autoimmune noninfectious uveitis through gene analysis [28]. In addition, fresh intraocular T cells from mice with EAU contained a large population of IL-22^+^cells, suggesting that Th22 cells may be associated with the pathogenic mechanisms of intraocular inflammation [29–31]. However, there are no studies on the biological features of IL-22 in the pathogenesis of uveitis and much remains to be explored about the role of IL-22 in EAU.

Cysteamine (2-aminoethanthiol) is currently used for the clinical treatment of nephritic cystinosis and used to treat cysteine crystal buildup in the cornea of patients with cystinosis [32, 33]. It has a strong antioxidant activity and has been implicated in the treatment of inflammation and neurodegenerative disorders [34–36]. In recent studies, cysteamine decreased the proliferation of PBMCs, the secretion of IL-6 and the TGF-β1 levels through ROS formation suggesting it targets inflammation-associated PBMCs that interact with corneal endothelial cells [37]. Because IL-6 is one of the inducers of IL-22 production, it suggests that cysteamine could also down-regulate IL-22 production.

Therefore, we examined whether the production of IL-22 and its receptor expression are increased in EAU and their role in the pathogenesis of EAU. Additionally, we investigated the anti-inflammatory effects of cysteamine in a murine model of EAU to determine whether cysteamine has a therapeutic potential effect on patients with uveitis by down-regulating IL-22 and its receptor.

## Materials and Methods

### Sample collection from patients with uveitis

The research performed in this study followed the tenets of the Declaration of Helsinki and was approved by the Institutional Ethics Committee of Seoul National University Hospital (protocol No.1503-027-654). After written informed consent was obtained, samples of serum and PBMCs were collected from 20 patients with a well-defined clinical diagnosis of acute and fresh uveitis without any medication and from 19 healthy donors as normal controls. The subjects were uveitis patients at the Boramae Medical Center and Seoul National University Hospital in Seoul, Korea. The healthy control subjects had no clinical history of uveitis or systemic diseases.

### Cell culture

Human retinal pigment epithelia cell line ARPE-19 was obtained from the American Type Culture Collection (ATCC) (Manassas VA, USA) and cultured in DMEM with 10% fetal bovine serum (GIBCO, Grand Island NY, USA), 100 U/ml of penicillin and 100 μg/ml of streptomycin in a humidified incubator at 37°C and 5% CO_2_.

### Confocal microscopy

ARPE-19 cells grown on coverslips were fixed with 4% PFA in PBS at 4°C. For immunocytochemistry staining, the ARPE-19 cells on the coverslips were incubated with 5% goat serum in 0.3% Triton X-100 in PBS at RT for 1 h. These cells were then incubated with rabbit anti-IL-22Rα (1:100; abcam, Cambridge, UK) overnight at 4°C. And then, the cells were incubated with Alexa fluor-633 conjugated anti-rabbit Ab (1:2,000; Invitrogen, Carlsbad CA, USA) for 60 min. at RT and finally stained with DAPI. The fixed eye samples were embedded in paraffin and stained with rabbit anti-IL-22Rα (1:200; abcam) and Alexa fluor-633 conjugated anti-rabbit Ab (1:2,000; Invitrogen).

### [^3^H]-Thymidine incorporation assay

ARPE-19 cells (5×10^3^) were seeded in 96-well flat plate with the presence of absence of rIL-22 (10 ng/ml). Result was the representative of three independent experiments and each experiment was performed in triplicate. After 30 hr, 1 μCi of [^3^H]-Thymidine (American Radiolabeled Chemicals, Inc., St. Louis, MO, USA) was added to each well. After 18 hr incubation, cells were harvested onto glass fiber filters using a cell harvester (Inotech biosystems international, Dietikon, Switzerland). When dry, these were sealed into polyethylene bags with scintillation fluid (BetaplateScint; PerkinElmer, Boston, MA, USA) and incorporated [^3^H]-thymidine counted on a MicroBeta Trilux 1450 (PerkinElmer).

### ELISA

ARPE-19 cells at 2×10^4^ were incubated for 48 h in the presence or absence of rIL-22 (10 ng/ml; R&D Systems). The concentration of TNF-α, IL-6, IL-8 and MCP-1 in the culture supernatants was measured by ELISA (BioLegend, San Diego CA, USA). The relative absorbance was measured at 450 nm using a microplate reader and the SoftMax Pro software (Molecular Devices, Sunnyvale CA, USA).

### Migration assay

A total of 5×10^4^ ARPE-19 cells suspended in serum-free media treated with or without rIL-22 (10 ng/ml) were placed in the lower compartment of a transwell migration chamber (Costar, Corning Inc., Corning NY, USA). The upper chamber with a 8 μm-pore transparent PET-filter was then coated with Matrigel Basement membrane matrix (BD Biosciences, San Jose CA, USA) and incubated at 4°C for 24 h. PBMCs at 1×10^5^ were then placed in the upper compartment, and cells were allowed to migrate for 72 h with conditioned media in the lower compartment in the presence or absence of anti-MCP-1 Ab (0.2 μg/ml) (R&D Systems, Minneapolis MN, USA). After 72 h, cells were visualized with Diftquick-staining solution (Sysmex, Chuo-ku, Kobe, Japan).

### Western blot

ARPE-19 cells at 5×10^5^ were incubated in the presence or absence of rIL-22 (10 ng/ml) for 5, 10, 20, and 30 min. The cells were then lysed, and the blocked membrane was incubated either with anti-pAkt Ab (1:1,000; Cell signaling Technology, Boston MA, USA), anti-Akt Ab (1:1,000; Cell signaling technology) or anti-β-actin (1:8,000; Sigma-Aldrich). The membrane was incubated with HRP-conjugated anti-rabbit IgG (1:15,000; Cell Signaling Technology) for p-Akt, Akt and HRP-conjugated anti-mouse IgG (1:10,000; Cell signaling technology) for β-actin for 1 h at RT.

### RT-PCR

To examine the signaling pathways for IL-22Rα expression, ARPE-19 cells at 3×10^5^ were pre-treated with a specific inhibitor for PI3K/Akt (LY294002; 10 μM; Sigma-Aldrich, St. Louis MO, USA) for 1 h. After rinsing with PBS, the cells were then cultured for 6 h in rIL-22 (10 ng/ml). The primer used for the RT-PCR was as follows: 5’-CCCCACTGGGACACTTTCTA-3’ (forward) and 5’-TGGCCCTTTAGGTACTGTGG-3’ (reverse) for IL-22Rα (243 bp); 5’-CATTGGGAATGGTACCAC-3’ (forward); and 5’-CCAATAATGGTGTCATCCAC-3’ (reverse) for IL-10Rβ (291 bp). To elucidate the correlation between IRBP_1-20_ and the IL-22Rα levels, cells at 3×10^5^ were treated with IRBP_1-20_ (1 μg/ml) for 1, 2, 4, and 6 h and collected at the indicated time points. In addition, the ARPE-19 cells were treated with or without IRBP_1-20_ and cysteamine (2.5 μg/ml) for 6 h.

### Mice EAU model with IRBP_1-20_ and treatment with cysteamine

Eight to ten-week-old female C57BL/6 mice weighing about 20 g each were purchased from Koatech (Pyeongtaek, Gyeonggi, Korea). The animals were handled and cared for according to the Association for Research in Vision and Ophthalmology (ARVO) guidelines for use of animals in ophthalmic and vision research and with the approval of the Institutional Animal Care and Use Committee (IACUC) of Seoul National University (Seoul, Korea). Human IRBP peptide sequence 1–20 (IRBP_1-20_) (PTHLFQPSLVLDMAKVLLD) was purchased from Peptron (Daejon, Chungchungnamdo, Korea). Purified Bordetella pertussis toxin (PTX) and cysteamine were purchased from Sigma-Aldrich, and complete Freund's adjuvant (CFA) and Mycobacterium tuberculosis strain H37Ra were purchased from Difco (Detroit MI, USA). Cysteamine was dissolved in PBS at 2 mg/ml and stored in aliquots at -80°C. To induce EAU, 250 μg of IRBP_1-20_ was emulsified in CFA (1:1 v/v) containing 2.5 mg/ml of M. tuberculosis H37Ra. A total of 100 μl of the emulsion was administered by injection into a footpad. Concurrent with immunization, 0.7 μg of PTX in 300 μl of PBS was injected intraperitoneally as an additional adjuvant.

### Cysteamine treatment

Starting one day before IRBP_1-20_ injection, cysteamine was administered daily and intraperitoneally at 40 mg/kg for 21 days. Control mice were administered intraperitoneally by injection of the same amount of PBS using the same schedule as the cysteamine-administered group. In the negative control group, neither IRBP_1-20_ nor cysteamine was injected into the mice.

### IL-22 levels in splenocytes with EAU and CD4+ T cells from uveitis patients

For IRBP_1-20_-specific T cell assay, the IRBP_1-20_-induced EAU mice were injected with cysteamine (40 mg/kg) intraperitoneally for 14 days. After the splenocytes were isolated from the IRBP_1-20_-injected mice treated or untreated with cysteamine, they were re-stimulated with IRBP_1-20_ (1 ug/ml) *in vitro*. After 24 hr, the supernatants from the cultures media were collected and assayed by ELISA for the detection of IL-22 levels. PBMCs were isolated from the blood samples of uveitis patients (n = 6) and healthy donors (n = 6). Using the CD4+ T cells isolation kit (130-096-533, Miltenyi Biotec, Auburn, CA, USA), human CD4+ T helper cells are isolated from PBMCs. The CD4+ T cells were cultured in the presence or absence of IRBP_1-20_ (1 μg/ml) and cysteamine (2.5 μg/ml) *in vitro* for 24 h. The culture supernatants were collected and used for the detection of IL-22 via ELISA.

### Evaluation of EAU

Clinical assessment of retinal inflammation was done with fundoscopy under a binocular microscope after dilation of the pupil. Fundoscopies were done every 3 or 4 days from day 10 after immunization following inoculation. Two ophthalmologists (T.W.K. and H.G.Y.) performed the clinical assessments in a masked fashion. The severity was graded on a scale of 0 (no disease) to 4 (severe disease) using a clinical criteria described elsewhere [5]. At 21 days after immunization, the mice were euthanized with CO_2_ gas. After excision, the eye tissues were processed for histopathological assessment. The higher score of the two eyes was used as the severity for each mouse.

### Statistical analysis

Data are presented as the means ± SEM. Statistical analysis of EAU scoring was done using the Mann-Whitney test. P values <0.05 were used to indicate a statistically significant difference (GraphPad Software, La Jolla, CA, USA).

## Results

### Differential serum IL-22 levels among uveitis patients and healthy donors

**Fig 1:**
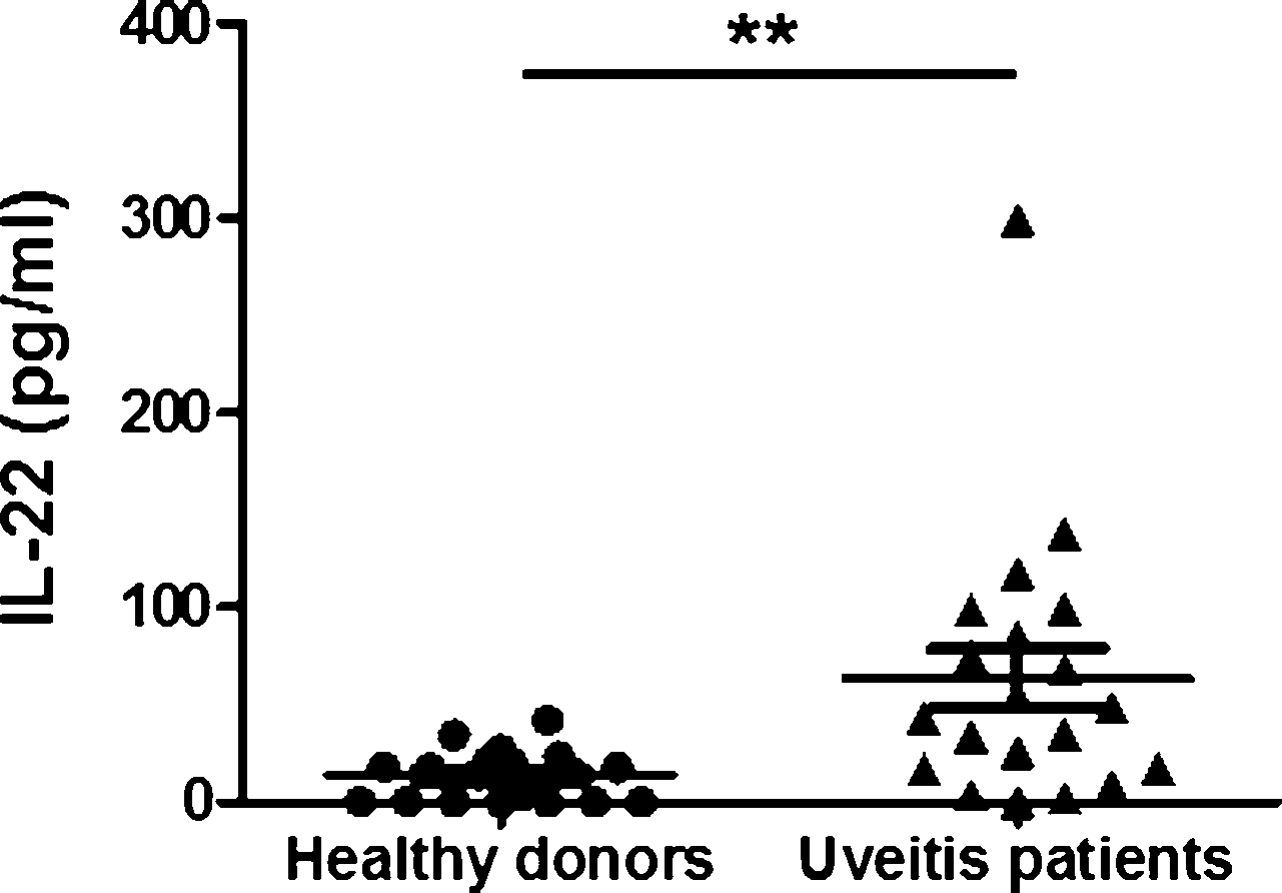
IL-22 serum levels in patients with uveitis. Serum was obtained from patients with uveitis (n = 20) and healthy donors (n = 19). It was separated from whole blood using a serum-separating tube by centrifuging at 600 x g for 10 min. IL-22 serum levels were measured with ELISA kits following the manufacturer’s instructions. *P-value* was obtained using unpaired two-tailed student’s t-test. ** p<0.001.

In our study, the serum IL-22 levels in the uveitis patients were significantly increased compared to those in the healthy controls (mean value ± SEM) (uveitis patients, n = 20; 63.35 ± 15.36 pg/ml *vs*. healthy controls, n = 19; 13.40 ± 3.002 pg/ml; *p = 0*.*0036*) (Fig 1A). Gender, age, and disease status are described in Table 1.

**Table 1:** Clinical information

	Healthy donors	Uveitis patients
**Number**	19	20
Age*	33 *ers	38 *ers p
**Sex**	Women: 10, Men: 9	Women: 10, Men: 10
**Clinical phase**	N/A	Acute
**Medication**	N/A	w/o any medication

*: mean ± SD

### Increased MCP-1 and proliferation of ARPE-19 cells by treatment with rIL-22

**Fig 2:**
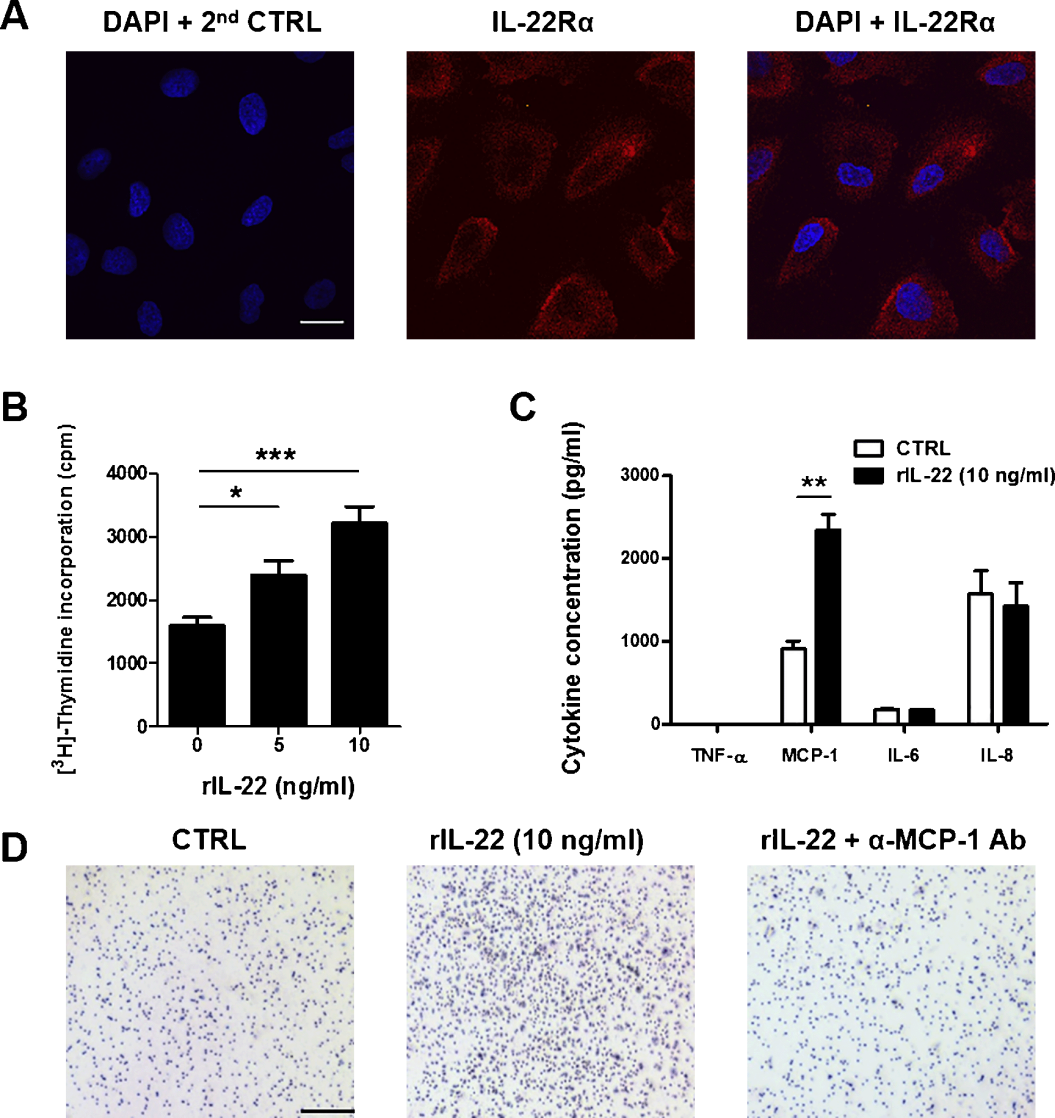
Production of MCP-1 and proliferation in ARPE-19 cells by treatment with rIL-22. (A) Expression of IL-22Rα in ARPE-19 cells was analyzed by confocal microscopy. Scale bar = 20 μm (B) ARPE-19 cells were treated with 5 ng/ml and 10 ng/ml of rIL-22 for 48 h. For the last 18 h, 1 μCi of [^3^H]-thymidine was added to each well. Each sample is in triplicates and results are representative of three independent experiments. (C) ARPE-19 cells were incubated with rIL-22 at 10 ng/ml for 24 h and the concentration of cytokines was measured with ELISA. (D) Migration of PBMCs in response to IL-22 was measured by the migration assay. ARPE-19 cells were placed in the lower chamber with ARPE-19 cells with or without rIL-22 and the upper chamber with PBMCs. PBMCs were then allowed to migrate for 72 h. Anti-MCP-1 Ab at 0.2 μg/ml was used for blocking MCP-1. Data were visualized with Diftquick-staining solution. Scale bar = 200 μm.* p<0.05, ** p<0.001, ***p<0.0001.

From the confocal microscope pictures, the expression of IL-22Rα was distributed throughout the cellular membrane and in the cytoplasm but not in the nucleus (Fig 2A). There was no staining in the secondary antibody only as a control. In the thymidine incorporation assay, recombinant IL-22 (10 ng/ml) induced a dose-dependent proliferation of ARPE-19 cells (control, 1592 ± 133.9 cpm; rIL-22 at 5 ng/ml, 2397 ± 228 cpm; and rIL-22 at 10 ng/ml, 3213 ± 263.3 cpm) (control *vs*. rIL-22 at 5 ng/ml, *p = 0*.*0251*) (control *vs*. rIL-22 at 10 ng/ml, *p = 0*.*0008*) (Fig 2B). To determine the effect of IL-22 on the production of inflammatory cytokines by ARPE-19 cells, the cells were cultured with rIL-22 (10 ng/ml) for 24 h. In the presence of rIL-22, ARPE-19 cells produced significantly higher amounts of MCP-1 than those cultured in the control medium (control; 904.2 ± 99.61 pg/ml *vs*. rIL-22-treated; 2347 ± 190.4 pg/ml; *p = 0*.*0026*). However, the rIL-22-treated ARPE-19 cells produced similar levels of TNF-α, IL-6 and IL-8 as those of the control cells (Fig 2C).

### Migration of PBMCs in response to IL-22

**Fig 3:**
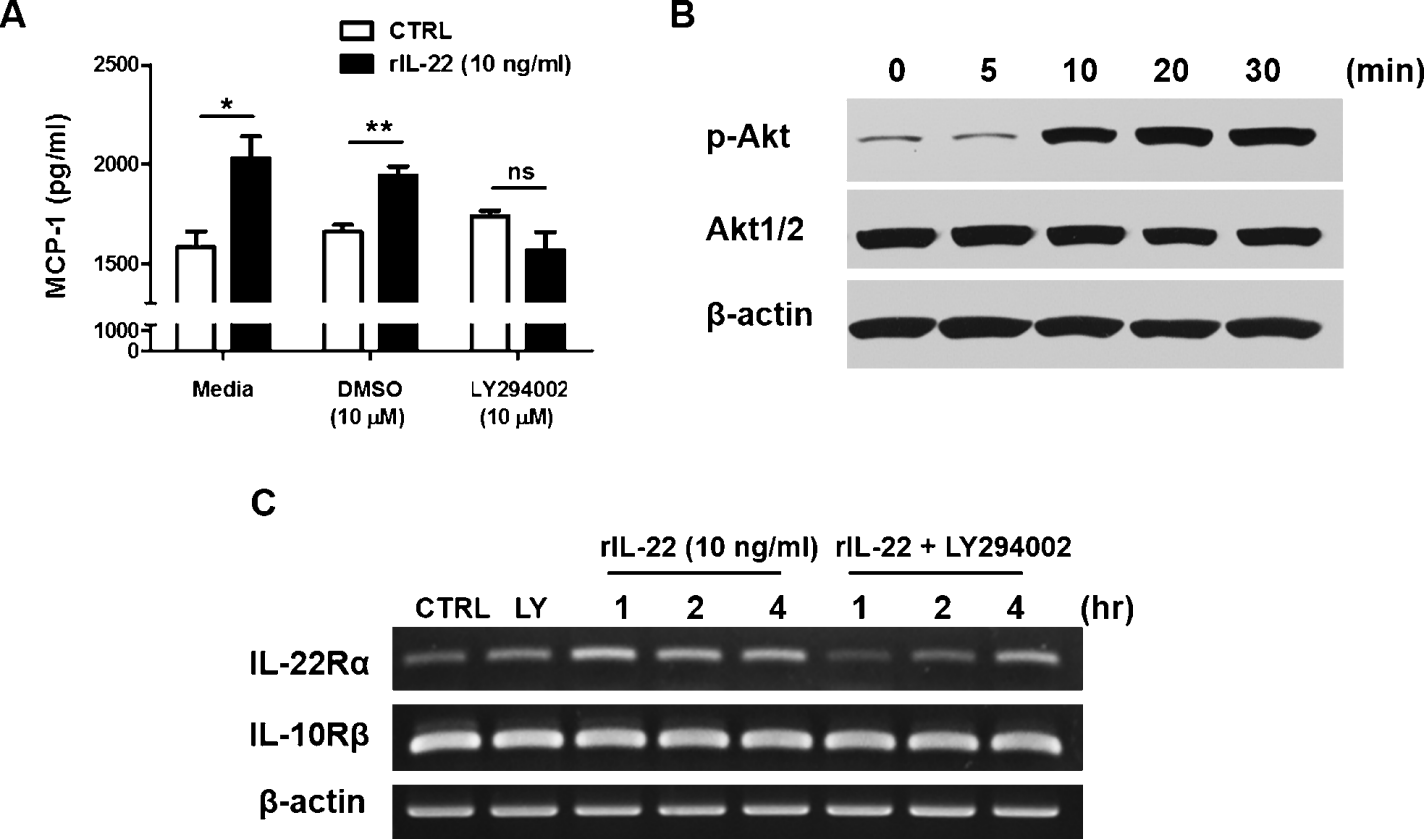
The effect of IL-22 on ARPE-19 cells through the regulation of its receptor expression. (A) ARPE-19 cells were pre-treated with DMSO (vehicle control) and LY294002 at 10 μM for 1 h and then cultured with rIL-22 at 10 ng/ml for another 24 h. (B) ARPE-19 cells were treated with rIL-22 at 10 ng/ml, and western blot for phosphorylation of Akt and Akt1/2 was performed as described in the Materials and Methods. (C) For mRNA expression, ARPE-19 cells were treated with rIL-22 alone or combined with LY294002 at 10 μM, an inhibitor of PI3K, and collected at the indicated time points, followed by RT-PCR.

To elaborate on the previous results, migration of PBMCs in response to rIL-22-treated ARPE-19 cells was measured using a migration assay. Because the blocking of MCP-1 using anti-MCP-1 Ab inhibited the migration of the PBMCs, it suggested that MCP-1 produced by ARPE-19 cells treated with rIL-22 drove the migration of PBMCs such as monocytes and lymphocytes (Fig 2D). According to the report by Murao K et al, PI3K/Akt pathway plays a crucial role in the production from vascular endothelial cells by TNF-α stimulation [38]. Therefore, an inhibitor study was carried out using LY294002, a potent PI3K inhibitor. As shown in Fig 3A, MCP-1 production from ARPE-19 was increased by rIL-22 treatment, but it was inhibited by pre-treatment of LY294002 (control treated with LY294002; 1738 ± 27.69 pg/ml *vs*. rIL-22 treated with LY294002; 1566 ± 92.81 pg/ml; *p = 0*.*1503*). It suggests that PI3K/Akt also plays a role in the production of MCP-1 by rIL-22 stimulation.

### Positive feedback amplification of IL-22Rα in ARPE-19 cells

Although IL-22 exerts its effect mainly though the activation of STAT-1, STAT-3, and STAT-5 along with the activation ERK-1/2 and p38 MAPK, a recent study has shown that IL-22 can also activate important kinases such as PI3/Akt. As shown in Fig 3A, PI3K inhibitor blocked the secretion of MCP-1; thus, PI3K/Akt signaling pathway may be involved in MCP-1 production. Because IL-22 led to the secretion of MCP-1, we speculated that IL-22 might lead to the activation of Akt. This is shown in Fig 3B in which phospho-Akt expression was increased after 10 min by stimulation with 10 ng/ml of rIL-22. In addition, RT-PCR was done with specific primers against IL-22Rα and IL-10Rβ. In Fig 3C, IL-22 in ARPE-19 led to an increased expression of IL-22Rα possibly through the activation of the PI3K/Akt pathway and phosphorylation of Akt. Because there was no change in the levels of IL-10Rβ, it suggests that IL-22 led to a positive feedback mediated through the increased expression of IL-22Rα.

### Hyperplasia of RPE and increased IL-22 production *in Vivo*

**Fig 4:**
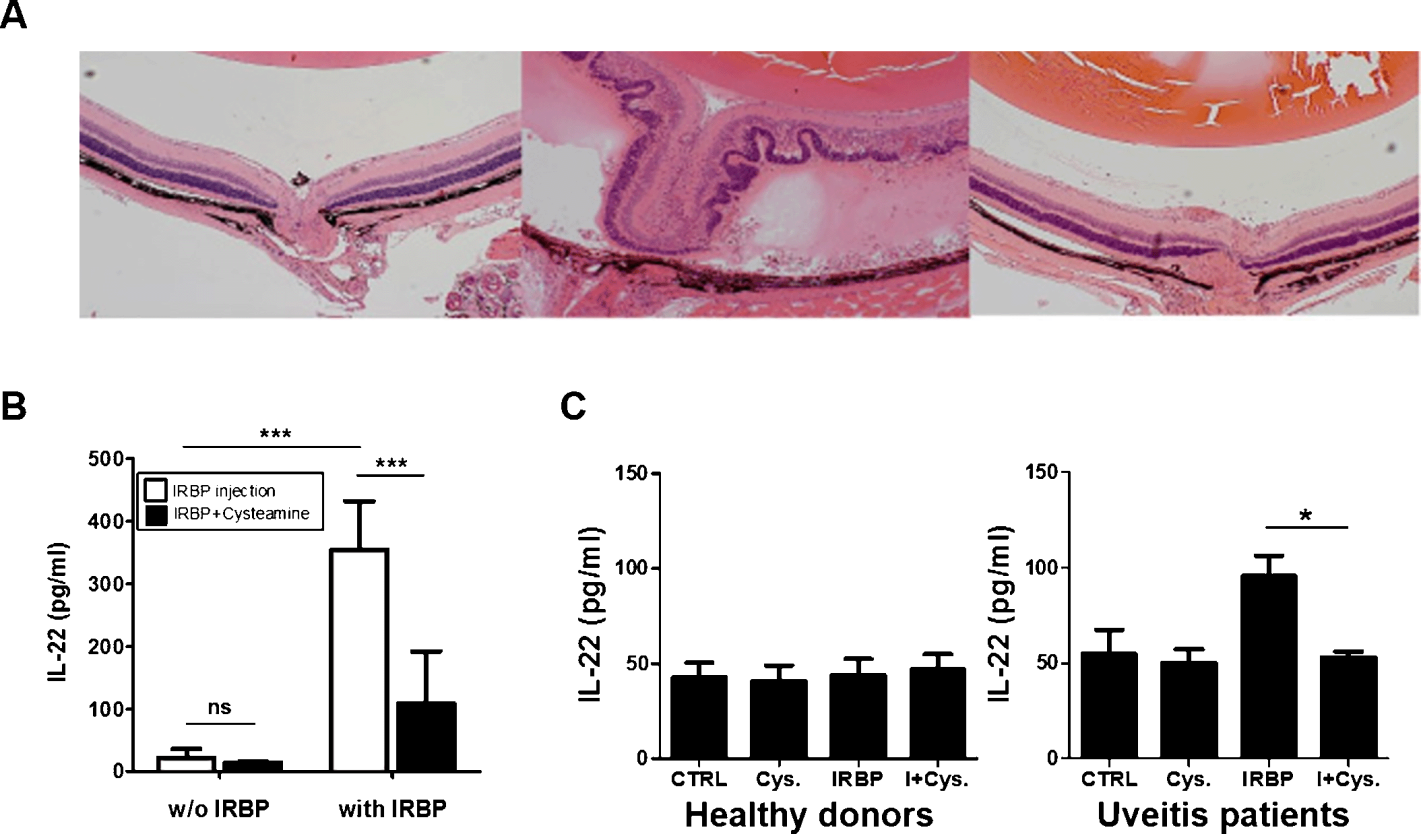
IL-22 production in IRBP_1-20_-induced EAU mice and uveitis patients. (A) Representative histopathology of mice treated with vehicle (PBS) alone or cysteamine. Negative control (left), mouse treated with PBS (middle), and mouse treated with cysteamine (right). On day 21 post immunization, the eyes from normal and EAU mice were enucleated and scored by examining the histopathological sections (H&E, x100 magnification). (B) Splenocytes from IRBP_1-20_-treated and IRBP_1-20_-treated with cysteamine injected mice were re-stimulated with IRBP_1-20_ (1 μg/ml) *in vitro* for 24 h. The supernatants from the cultures were collected and assayed for IL-22 by ELISA. *P-value* was obtained with unpaired two-tailed student’s t-test. *** p<0.0001 (C) IL-22 levels in the CD4+ T cells of uveitis patients (n = 6) and healthy control (n = 6). *P-value* was obtained with unpaired two-tailed student’s t-test. * p<0.05.

Next, the potential anti-inflammatory effect of cysteamine was investigated in IRBP_1-20_-induced EAU in C57BL/6 mice. Left microscopy of ocular sections showed normal-appearing retina, and there was no systemic toxicity (Fig 4A). Inflammatory cells along with retinal folds and granulomatous lesions and hyperplasia of RPE were more severe in the retina, vitreous and choroid of the IRBP_1-20_-induced EAU mice compared to the cysteamine-treated EAU mice at 21 days post immunization. To examine whether cysteamine treatment could alter the production of pro-inflammatory cytokines such as IL-22, the IL-22 level was quantified by IRBP_1-20_-specific T cells assay. The level of IL-22 by ELISA was significantly increased in IRBP_1-20_-treated splenocytes compared to control non-treated cells. However, cells that were treated *in vivo* with cysteamine showed a significantly decreased production of IL-22 compared with those that were not treated with cysteamine (Fig 4B). In addition, PBMCs were isolated from uveitis patients and healthy donors. In Fig 4C, cysteamine treated PBMCs from the uveitis patients had lower IL-22 levels than those treated with IRBP_1-20_ (Fig 4C).

### Down-regulation of IL-22Rα by cysteamine *in Vitro* and *in Vivo*

**Fig 5:**
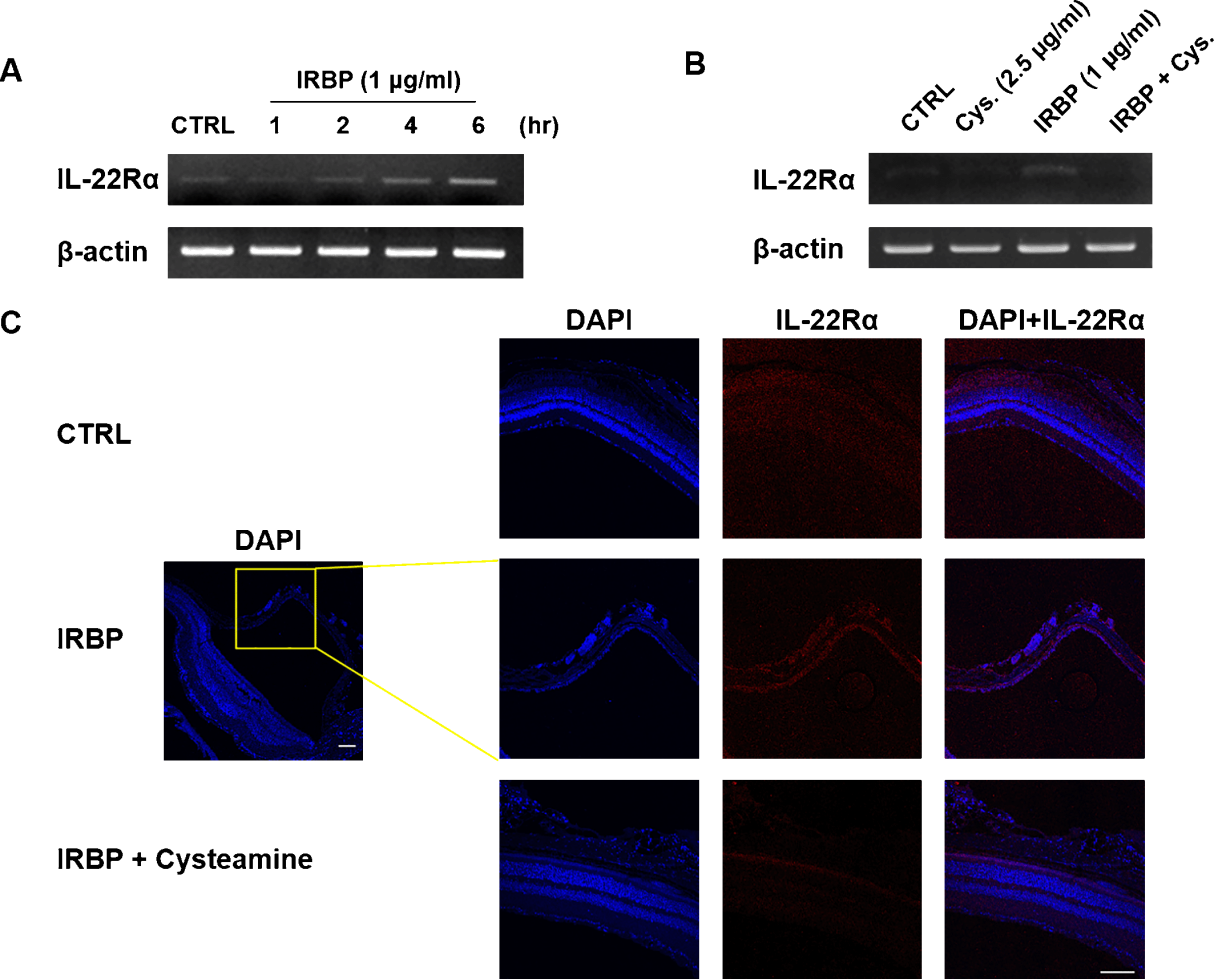
The regulation of IL-22Rα expression in ARPE-19 cells treated with cysteamine. (A) Cells were treated with IRBP_1-20_ at 1 μg/ml for 1, 2, 4, and 6 h and collected for RT-PCR analysis at the indicated time points. (B) Cells were cultured with or without IRBP_1-20_ and cysteamine at 2.5 μg/ml for 6 h. RT-PCR was performed as described in the Materials and Methods. (C) Eye tissues isolated from each mouse (control *vs*. IRBP_1-20_-induced EAU model *vs*. IRBP_1-20_-induced EAU model treated with cysteamine) were fixed in 4% PFA at 4°C overnight. Fixed eye tissues were embedded in paraffin and sectioned. The expression of IL-22Rα in eye tissue was assessed by confocal microscopy. Scale bar = 100 μm.

Because IL-22Rα expression was shown to be upregulated in the liver of concanavalin A injected mice in a previous study, [25] we investigated whether IRBP_1-20_ and cysteamine regulated the expression of IL-22Rα in ARPE-19 cells. IRBP_1-20_ upregulated IL-22Rα expression in ARPE-19 cells in a time-dependent manner (Fig 5A). In addition, the increased expression of IL-22Rα was down-regulated by treatment with cysteamine in ARPE-19 cells (Fig 5B). Moreover, IRBP_1-20_ upregulated IL-22Rα expression in retinal pigmented epithelium and induced retinal detachment. However, the downregulation of IL-22Rα expression was observed in the eyes of cysteamine-treated mice. Therefore, the effect of cysteamine on ARPE-19 cells observed earlier could be through the down-modulation of IL-22Rα levels (Fig 5C).

### Attenuation of the severity in EAU by treatment with cysteamine

**Fig 6:**
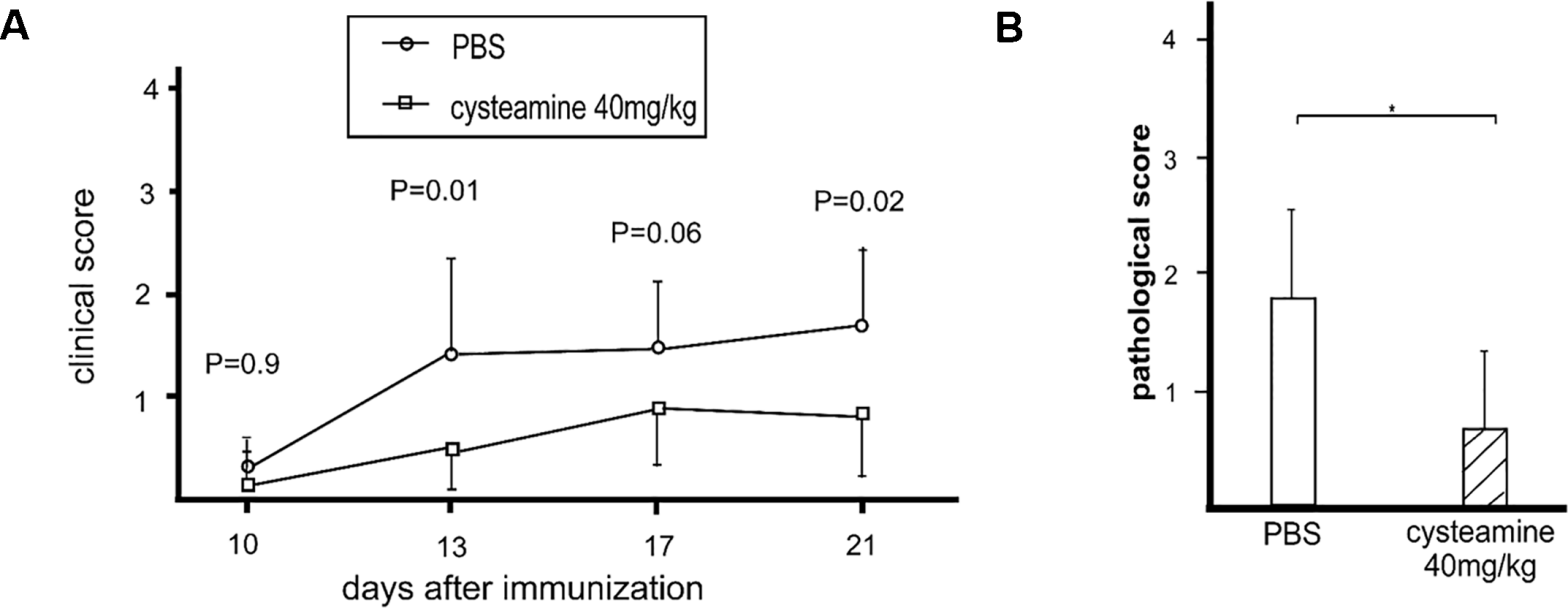
The effect of cysteamine on EAU. (A) A diagram of clinical scoring of IRBP_1-20_-induced uveitis. Clinical score of EAU in mice treated with vehicle (PBS, n = 21) alone or cysteamine (40 mg/kg) (n = 18). EAU was induced as described in the Materials and Methods. Results are presented as the mean clinical score for all eyes for each group of mice, and the significance was determined with the Mann-Whitney test. (B) A diagram of histopathology scores of IRBP_1-20_-induced uveitis. Histopathological score of EAU was determined by histopathology on day 21 post immunization. Each symbol represents one mouse, showing the higher score of the two eyes for each mouse. Mean EAU score of each group is indicated by a bar. The pathological scores of retinal sections were significantly lower in the cysteamine-treated group (n = 27) than in the control group (n = 26). Results are expressed as the mean ± standard deviation, and significance was determined with the Mann-Whitney test. * p<0.05.

Next, clinical grading was carried out every 3 or 4 days for 10 days after immunization. Clinical examinations were performed in the control (PBS treated) and cysteamine treated (40 mg/kg) groups. In the control group (n = 21), vascular sheathing and multiple yellow dots were evident in the fundus at day13. For the cysteamine-treated group (n = 18), the fundoscopic examination showed mild vascular sheathing at 13 and 21 days post immunization (control *vs*. cysteamine; at 13 days, *p = 0*.*01;* and at 21 days, *p = 0*.*02*) (Fig 6A).

Disease onset was significantly delayed in the cysteamine-treated group compared with that in the control group (control; 12.1 ± 1.4 days *vs*. cysteamine; 14.6 ± 3.0; *p = 0*.*02*, Mann-Whitney test). The histological score in mice treated with cysteamine was also significantly lower than that in the control mice (control *vs*. cysteamine; *p = 0*.*03*) (Fig 6B). Taken together, cysteamine might be a useful therapeutic drug for uveitis by down-regulating IL-22Rα expression in RPE.

## Discussion

Interestingly, we found that IL-22Rα expression is increased in ARPE-19 by stimulation with IRBP_1-20_, and its expression was inhibited by pre-treatment with cysteamine, even though it is known that IRBP_1-20_ does not have a stimulatory effect on ARPE-19 [1, 39]. However, we found the same results after repeating the experiment more than five times. Thus, we did a limulus assay to check for LPS contamination in our experimental materials, such as the PBS and media; however, there was no LPS contamination. Because IRBP_1-20_ is a short peptide that can be degraded very quickly in the serum and culture media with FBS, the experiment on the stimulatory effect of IRBP_1-20_ in ARPE-19 was done with relatively high doses of IRBP_1-20_ within a short period. Therefore, we believe that this is the reason why we could detect the stimulatory effects of IRBP_1-20_.

The RPE is a monolayer of cells situated between the neuroretina and choroid. The RPE can contribute to either immunosuppressive or inflammatory responses in the eye through the secretion of cytokines, antagonists, and soluble cytokine receptors. Of interest, IL-6, IL-8, TNF-α, and MCP-1 are secreted to the choroidal side of the RPE layer in a polarized fashion [40–42]. Polarized secretion of these cytokines has an important role in immune processes in the posterior part of the eye. Inflammatory cell infiltration in the eye and secretion of inflammatory cytokines lead to intraocular inflammation that can ultimately cause blindness [43].

Pathologically, macrophages have a crucial role in the initial phase of uveitis [44, 45]. Recruited macrophages can exert their inductive functions on the development and persistence of inflammation through the production of NO and phagocytosis of rod cells and cone cells in the retina. This aggravates the inflammation of the posterior segment in the eye [46]. In addition, IL-22 from Th17 T cells may have an important role in the inflammatory scenario of uveitis because IL-22 also induces MCP-1 production in autoimmune settings such as rheumatoid arthritis [22]. MCP-1 gene transcription is upregulated in response to TNF-α in vascular endothelial cells through the PI3K/Akt [38]. As shown in Figs 2C and 3A, RPE may direct lymphocyte and monocyte migration during posterior uveitis through CC chemokines.

IL-22 activates the three major MAP kinase pathways JNK, p38 MAPK, and ERK-1/2 [9, 47]. IL-22 induces the proliferation of normal human epidermal keratinocytes obtained from healthy individuals and fibroblasts like synoviocytes isolated from psoriatic arthritis, rheumatoid arthritis and osteoarthritis patients [22, 48–50]. In our study, ARPE-19 cells were pre-treated with specific inhibitors for ERK (PD98059), JNK (SP600125), PI3K/Akt (LY294002), p38 MAPK (SB203580), and NK-κB (Bay11-7082) for 1 h. When the ARPE-19 cells were treated with rIL-22, we were able to observe p38MAPK and NK-κB dependent proliferation of ARPE-19 cells (S1 Fig).

Taken together, we have not only found that IL-22 production and its receptor expression are closely related with the development and pathogenesis of uveitis through the increase of MCP-1 production followed by the induction of inflammatory cell infiltration into the ocular lesion, but also found that cysteamine effectively prevented this process in the present study. Because the disease is characterized by its persistence and recurrence, endogenous uveitis could be considered as a social and economic burden. If an effective cure is indeed found, its economic value is believed to be very large.

## Supporting Information

S1 Fig P38 MAPK and NK-κB dependent proliferation of ARPE-19 cells treated with rIL-22. (TIF)

S1 File Supplementary Materials. (PDF)
