# Supplementary figures and images for: The Role of Interleukin-22 and Its Receptor in the Development and Pathogenesis of Experimental Autoimmune Uveitis
Source: PLoS One. 2016 May 11;11(5):e0154904. doi: 10.1371/journal.pone.0154904 (PMC4864334; doi:10.1371/journal.pone.0154904)

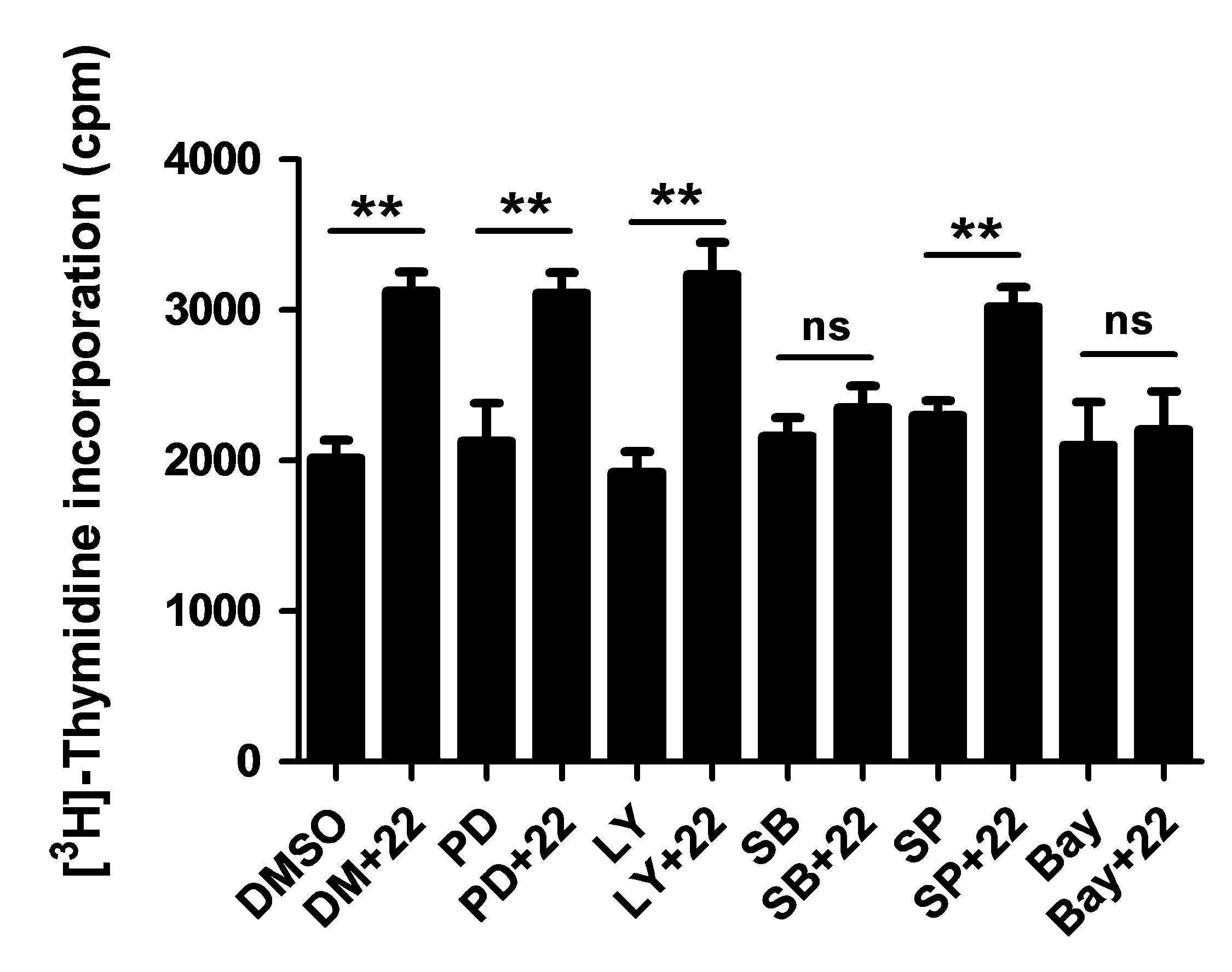

Supplement: S1 Fig — (TIF) [file pone.0154904.s001.tif]
