# Supplementary material for: The Role of Interleukin-22 and Its Receptor in the Development and Pathogenesis of Experimental Autoimmune Uveitis
Source: PLoS One. 2016 May 11;11(5):e0154904. doi: 10.1371/journal.pone.0154904 (PMC4864334; doi:10.1371/journal.pone.0154904)
Supplement: S1 File — (PDF) [file pone.0154904.s002.pdf]

S1 Fig.

Materials and methods

[^{3}H]-Thymidine incorporation assay

ARPE-19 cells at 5×10^3 were seeded in a flat-bottom 96-well plate with the specific inhibitors ERK (PD98059; 20 μM; Sigma-Aldrich), JNK (SP600125; 20 μM; Sigma-Aldrich), PI3K/Akt (LY294002; 10 μM; Sigma-Aldrich), p38 MAPK (SB203580; 10 μM; Sigma-Aldrich), and NK-κB (Bay11-7082; 5 μM; Sigma-Aldrich) for 1 h. After rinsing with PBS, cells were cultured in the presence or absence of rIL-22 (10 ng/ml). After 30 h, 1 μCi of [^{3}H]-Thymidine (American Radiolabeled Chemicals) was added to each well. After an18 h incubation, cells were harvested onto glass fiber filters using a cell harvester (Inotech Biosystems International). When dry, these were sealed into polyethylene bags with scintillation fluid (BetaplateScint), and incorporated [^{3}H]-thymidine was counted on a MicroBeta Trilux 1450 (PerkinElmer).
